# Mesenchymal stem cells can improve discogenic pain in patients with intervertebral disc degeneration: a systematic review and meta-analysis

**DOI:** 10.3389/fbioe.2023.1155357

**Published:** 2023-06-16

**Authors:** Wupeng Zhang, Daofeng Wang, Hua Li, Gaoxiang Xu, Hao Zhang, Cheng Xu, Jiantao Li

**Affiliations:** ^1^ School of Medicine, Nankai University, Tianjin, China; ^2^ Senior Department of Orthopedics, The Fourth Medical Center of Chinese PLA General Hospital, Beijing, China; ^3^ National Clinical Research Center for Orthopedics, Sports Medicine and Rehabilitation, Beijing, China

**Keywords:** mesenchymal stem cells, intervertebral disc, degeneration, lumbar discogenic pain, meta-analysis

## Abstract

**Background:** The meta-analysis aimed to estimate the efficacy of mesenchymal stem cells on lumbar discogenic pain in patients with intervertebral disc degeneration.

**Methods:** A comprehensive literature search was conducted in the PubMed, Web of Science, Embase and Cochrane Library databases with predetermined search strategy up to 18 September 2022. The clinical studies focusing on evaluating the efficacy and safety of mesenchymal stem cells in patients with intervertebral disc degeneration were identified. The primary outcomes were changes of pain score and Oswestry Disability Index. The Newcastle-Ottawa Scale for cohort studies was used for quality assessment. Review Manager was used to conduct the statistical analysis. Pooled risk ratios were calculated based on the random effect model. Heterogeneity, subgroup, and publication bias analyses were also performed.

**Results:** There were 2,392 studies were identified in the initial search, and 9 eligible studies with 245 patients were eventually included in this review. The Visual Analogue Scale score was significantly lower in patients after receiving mesenchymal stem cells therapy (mean difference = 41.62; 95% confidence interval 24.32 to 58.93; Heterogeneity: I^2^ = 98%; *p* < 0.01). And the pooled mean difference of Oswestry Disability Index was 22.04 from baseline to final follow-up points (95% confidence interval 8.75 to 35.33; *p* = 0.001; Heterogeneity: I^2^ = 98%; *p* < 0.001). The pooled reoperation proportion was 0.074 (95% confidence interval 0.009 to 0.175; Heterogeneity: I^2^ = 72%; *p* < 0.01). There were no serious related adverse events associated with the therapy.

**Conclusion:** The findings of this meta-analysis indicated that mesenchymal stem cells therapy may be effective in relieving pain and improving Oswestry Disability Index significantly in patients with lumbar discogenic pain. Mesenchymal stem cells therapy may also be associated with a lower risk of adverse events and reoperation rates.

## 1 Introduction

A significant increase in the morbidity and disability of low back pain led to a serious global economic burden, the total cost of low back pain in the United States in 2006 was estimated exceed $100 billion, it is widely accepted that intervertebral disc (IVD) degeneration is the central pathogenesis of pain in patients experiencing low back pain (LBP) (Urban and Roberts, 2003; Katz, 2006; Hurwitz et al., 2018; Dieleman et al., 2020). Intervertebral disc degeneration is commonly prevalent in people over the age of 50 (Teraguchi et al., 2014), and over 80% of adults will experience low back pain at some stage in their lives. The risk of degeneration is higher in men than in women and is more common in the lumbar spine confirmed by medical images (Jarraya et al., 2018). At present, non-surgical treatment based on physiotherapy and pharmacological interventions remains the first-line treatment option for lumbar discogenic pain. In recent years, mesenchymal stem cells (MSCs) intervertebral disc injection therapy has been used as an alternative treatment to stop or reverse the degenerative process and restore the functionality of the IVD (Elabd et al., 2016; Noriega et al., 2017; Atluri et al., 2022; Bates et al., 2022).

The use of cell-based therapies has been discussed as an alternative treatment option for intervertebral disc degeneration, potentially repopulating and repairing damaged discs and regulating the degenerative environment. According to relevant studies, Mesenchymal stem cells (MSCs) are primarily used for tissue repair through paracrine expression and cell-to-cell interactions (Caplan and Correa, 2011; Spees et al., 2016). MSCs have been shown to be a promising candidate source of cells for the treatment of degenerative intervertebral disc diseases due to their easy accessibility in bone marrow, adipose, synovium, periosteum, and cartilage. Multiple published studies showed clinically meaningful improvement in pain and disability in patients with lumbar disc degeneration and chronic low back pain (LBP) within 1 year of injection of mesenchymal stem cells (Orozco et al., 2011; Kumar et al., 2017; Noriega et al., 2017).

Although several clinical trials have been conducted to demonstrate that MSCs can stop or even reverse the progression of disc disease and have become one of the main therapeutic strategies for treating patients with discogenic pain, trial results have often hampered by sample size and experimental design, which prevent us from fully understanding the therapeutic efficacy of MSCs and collecting strong evidence of the efficacy and safety of stem cell therapy. Given that the present systematic review and meta-analysis of clinical studies, aiming to evaluate the efficacy of MSCs against intervertebral disc (IVD) degeneration in patients with lumbar discogenic pain.

## 2 Methods

This systematic review and meta-analysis conducted in accordance with the Preferred Reporting Items for Systematic reviews and Meta-Analyses (PRISMA) Statement protocol (Shamseer et al., 2015; Cumpston et al., 2019).

### 2.1 Search strategy and eligibility criteria

PubMed, Web of Science, Embase and Cochrane Library databases were searched through on 18 September 2022. The key search terms were (intervertebral disc degeneration OR low back pain) AND (Mesenchymal Stem Cells). We developed concrete search strategies for each database, for example, by combining key search terms and MeSH or EMTREE, and the potential eligibility of the identified studies was checked by inclusion criteria and exclusion criteria.

The following inclusion criteria identified eligible publications:

Publications focusing on the efficacy and safety of MSCs in the treatment of discogenic pain in patients with intervertebral disc degeneration.

Clinical trials or influential clinical studies.

The main observations included changes in pain scores and the Oswestry Disability Index (ODI) after treatment.

After the removal of duplicates, and then we excluded non-English language reports, *in vitro* studies, basic experimental studies, case reports, brief reports, conference abstract/posters, presentations or reviews. The reference lists were screened by two authors independently according to screening criteria, with the titles and abstracts reviewed to screen for potentially eligible studies. All relevant full-text papers were then scrutinized and assessed independently by the same two reviewers to determine the final list of publications that meet the eligibility criteria for the current study. In the event of a discrepancies occurred, a third senior author was consulted for final assessment and consensus. The full search strategy of each database is shown in the Supplemental Table S1.

### 2.2 Data extraction

After the final list of included studies was determined, data were extracted into a pre-built data sheet, including information on title, author, region, year published, age, gender, sample size, mesenchymal stem cell type, follow-up period, and main evaluation index and study design. The primary outcome was changes of pain relief and Oswestry Disability Index (ODI). Adverse events and reoperation proportion were also extracted as secondary outcomes. If the necessary information could not be extracted from the original paper, we contacted the corresponding author to obtain the relevant information.

### 2.3 Assessment of quality and bias

Two reviewers assessed the quality of the included studies independently. In this meta-analysis, the modified Jadad Scale was employed for RCTs (Moher et al., 1996), and the Newcastle-Ottawa Scale (NOS) for cohort studies were used for quality assessment (Stang, 2010). Ranges of NOS scale scores from 0 to 9, with scores of 7 and above considered to be of high quality. The funnel plot was used for estimating the publication bias. When disagreement occurred, a third senior orthopedic surgeon was consulted for final consensus.

### 2.4 Statistical analysis

Statistical analysis was performed by Cochrane Review Manager (RevMan) version 5.3, with value of *p* < 0.05 as statistically significant. For continuous data with standard deviation, meta-analysis was performed to calculate the weighted mean difference (WMD) with 95% confidence intervals (CI). When comparing the incidence of dichotomous data, such as rate of reoperation, risk ratio (RR) was calculated with the confidence intervals (CI). We used the Higgins I-squared (I^2^) statistic and Q test to measure heterogeneity. If I^2^ < 50% and the *p*-value for Q test was > 0.05, then the studies were interpreted as minimally heterogeneous and a fixed-effects model was applied for the meta-analysis. A random-effects model was applied when I^2^ > 50% or the *p*-value for the Q test < 0.05, which indicated that the data were of considerable heterogeneity. Other descriptive results suitable for quantitation were presented as a descriptive summary. Other descriptive results suitable for quantitation were presented as a descriptive summary. Subgroup-analysis were applied for significant heterogeneity.

## 3 Results

### 3.1 Overview of search results

**FIGURE 1:**
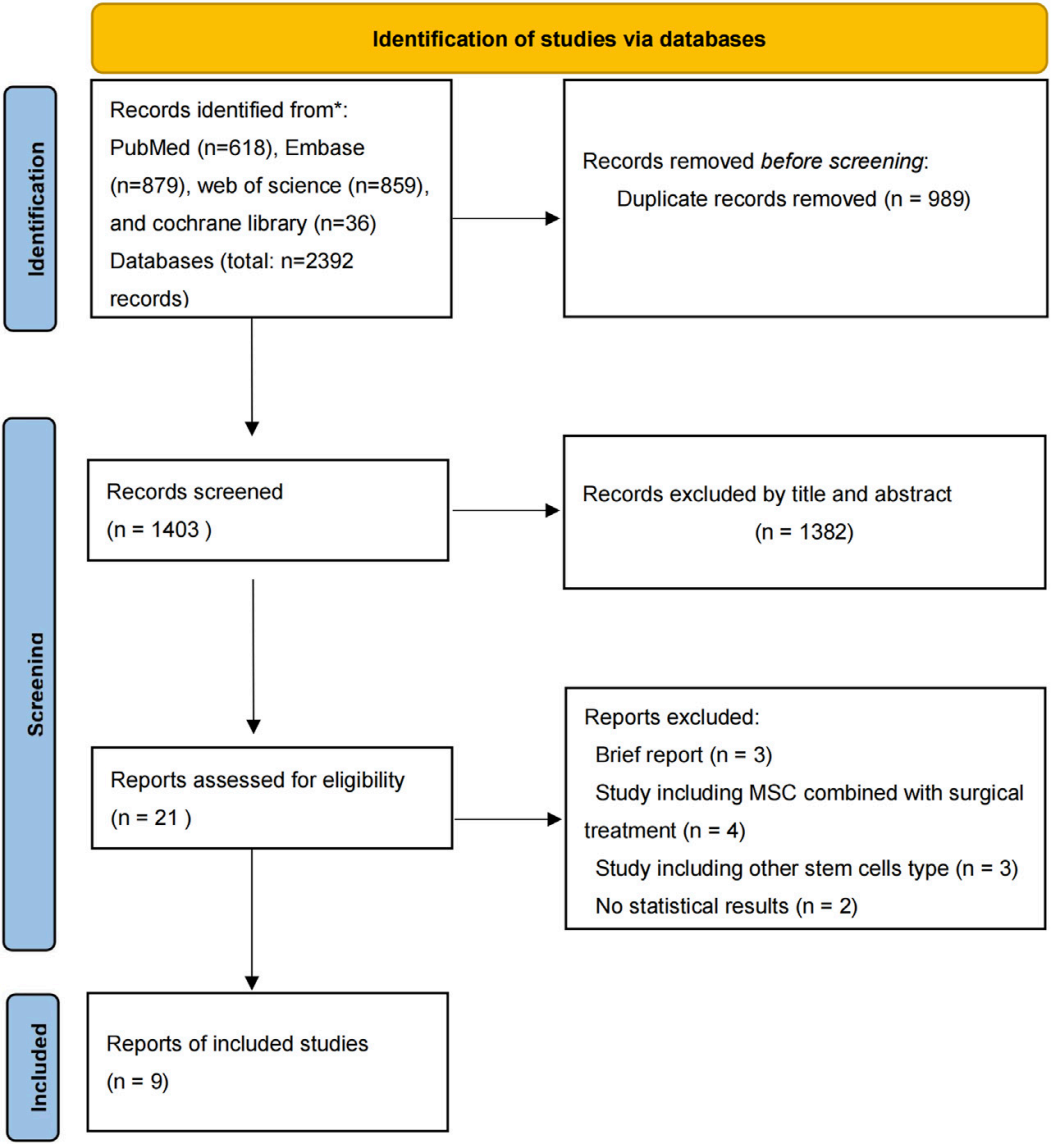
PRISMA diagram: summary of literature search results.

There were 2,392 studies were identified in the initial search. The titles and abstracts were then reviewed for 1,403 papers after dropping duplications. There were 21 publications further assessed by full-text reading for eligibility. Finally, 1 RCT and 8 prospective cohort studies published from 2011 to 2022 were included in the eventual analysis (Orozco et al., 2011; Pettine et al., 2014; Pettine et al., 2015; Centeno et al., 2017; Kumar et al., 2017; Noriega et al., 2017; Pettine et al., 2017; Papadimitriou et al., 2021; Atluri et al., 2022) (Figure 1). All of these studies have been published in the last 12 years. There were 245 patients eventually included in this review and 193 patients who received MSCs intervertebral disc injection therapy. The details of included studies were summarized in Table 1.

**TABLE 1:** Characteristics of included studies.

Authors	Year	Study design	Sample size (M/C) (n)	VAS	ODI	Reoperation proportion (n)	Fellow up period(y)	AE(n)
Pettine et al	2014	cohort study	26	-	-	2/26	1	0
Noriega et al	2016	RCT	12/12	67 ± 7→	34 ± 7→	0/12	1	0
				47 ± 10	22 ± 10			
Pettine et al	2017	cohort study	26	82.1 ± 2.6→	56.7 ± 3.6→	6/26	3	0
				21.9 ± 4.4	17.5 ± 3.2			
Sairam et al	2022	cohort study	40/40	-	46.1 ± 12.6→	0/40	1	0
					31.1 ± 18.9			
Orozco et al	2011	cohort study	10	68.9 ± 3.3→	25.0 ± 4.1→	0/10	1	0
				20.0 ± 6.5	7.4 ± 2.3			
Kumar et al	2017	cohort study	10	65 ± 12.7→	42.8 ± 15→	0/10	1	0
				29 ± 16.6	16.8 ± 9.8			
Centeno et al	2017	cohort study	33	-	-	2/33	6	0
Pettine et al	2015	cohort study	26	-	-	5/26	2	0
Papadimitriou et al	2021	cohort study	10	-	-	5/10	2	0

M/C, Mesenchymal Stem Cell group/Control group; n, number; RCT, randomized control trial; AE, adverse events; y, year.

### 3.2 Assessment of quality and bias

**FIGURE 2:**
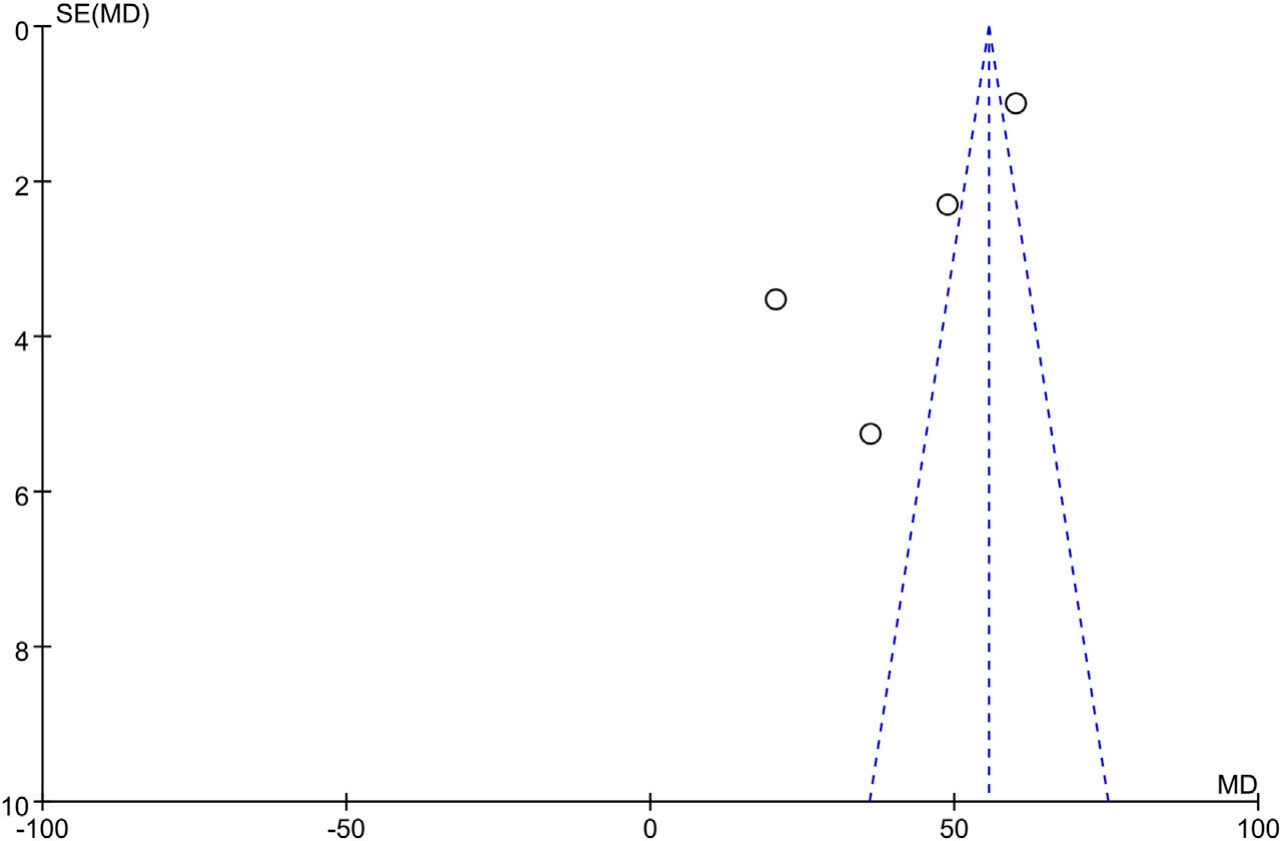
Funnel plot of VAS publication bias.

**FIGURE 3:**
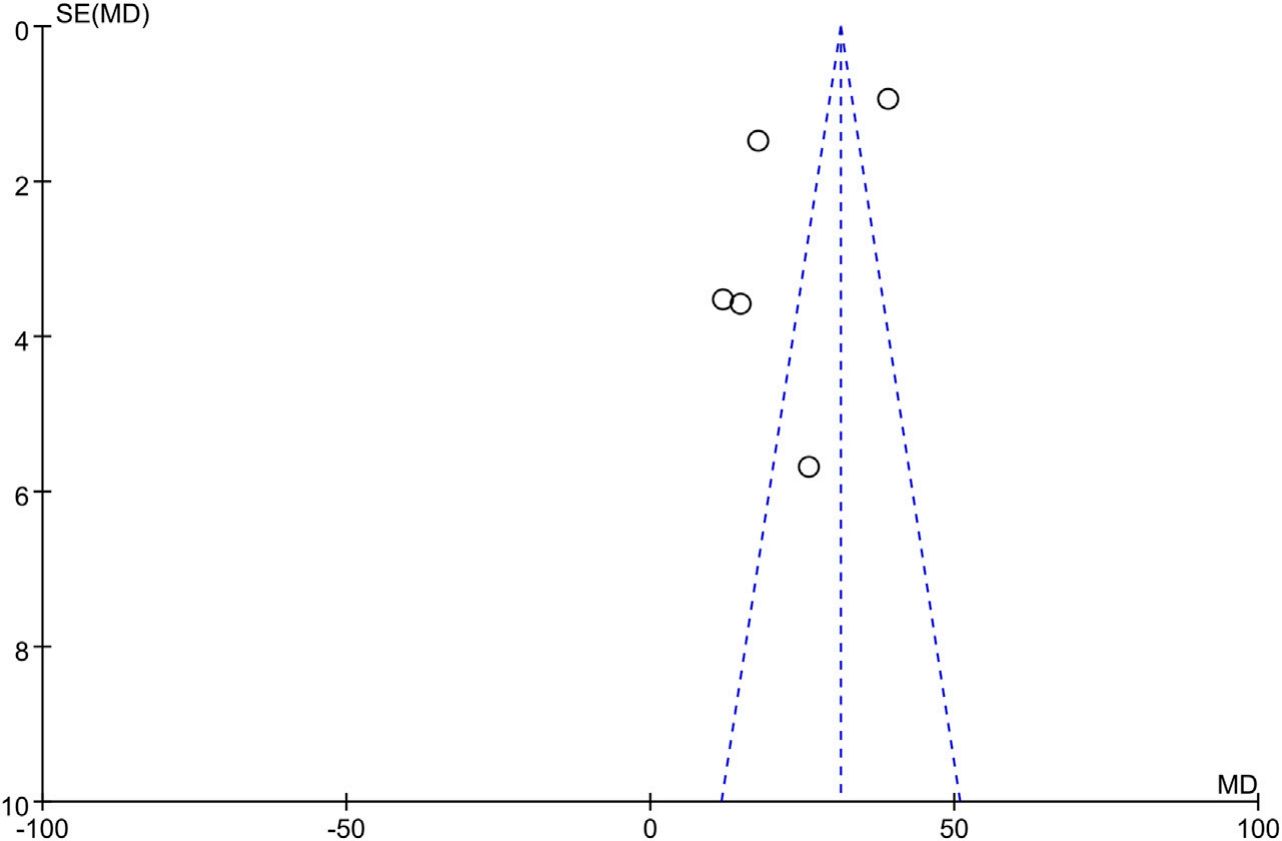
Funnel plot of ODI publication bias.

The only RCT included in this study had a Jadad score above 4 (Randomization: 1 scores, Concealment: 1 scores, Blinded: 2 scores, and Withdraw or drop-out: 1 scores), which indicates a high quality. The Newcastle-Ottawa was assessed for 8 high quality cohort studies (Table 2). Studies scoring 4–6 and 7 or more were classified as medium and high quality respectively. Overall, the funnel plot did not show the concerns of possible publication bias of Visual Analogue Scale (VAS) and ODI. (Figure 2; Figure 3).

**TABLE 2:** Quality assessment by the Newcastle-Ottawa Scale for cohort study.

Study	Selection				Comparability	Outcome		
	1	2	3	4	1	1	2	3
Pettine et al., 2014	+	+	+	+	+	+	-	+
Pettine et al., 2017	+	+	+	+	+	+	-	+
Sairam et al	+	+	+	+	+	+	-	+
Orozco et al	+	+	+	+	+	+	-	+
Kumar et al	+	+	+	+	+	+	-	+
Centeno et al	+	+	+	+	+	+	+	+
Pettine et al., 2015	+	+	+	+	+	+	-	+
Papadimitriou et al	+	+	+	+	+	+	-	+

Six or more “+” represented a high-quality study.

### 3.3 Primary outcome

**FIGURE 4:**
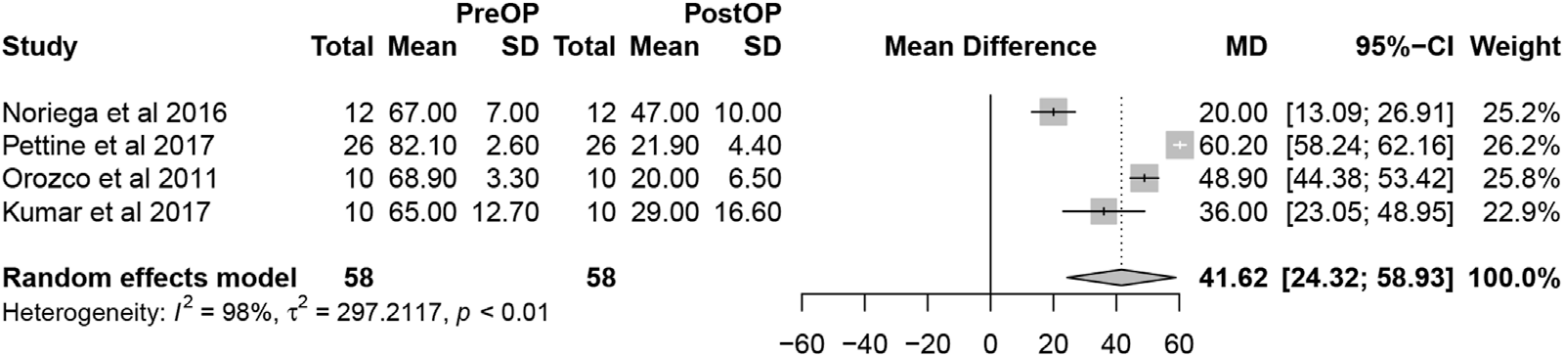
Forest plot for the VAS.

Overall, the detailed VAS data were reported in 4 studies. The pooled analysis of 4 studies demonstrated that MSCs therapy could significantly decrease VAS scores (mean difference = 41.62; 95% confidence interval [CI] 24.32 to 58.93 Heterogeneity: I^2^ = 98%; *p* < 0.01) (Figure 4). The remaining 5 studies did not offer the data with the standard deviation or only reported the percentage of score improvement.

**FIGURE 5:**
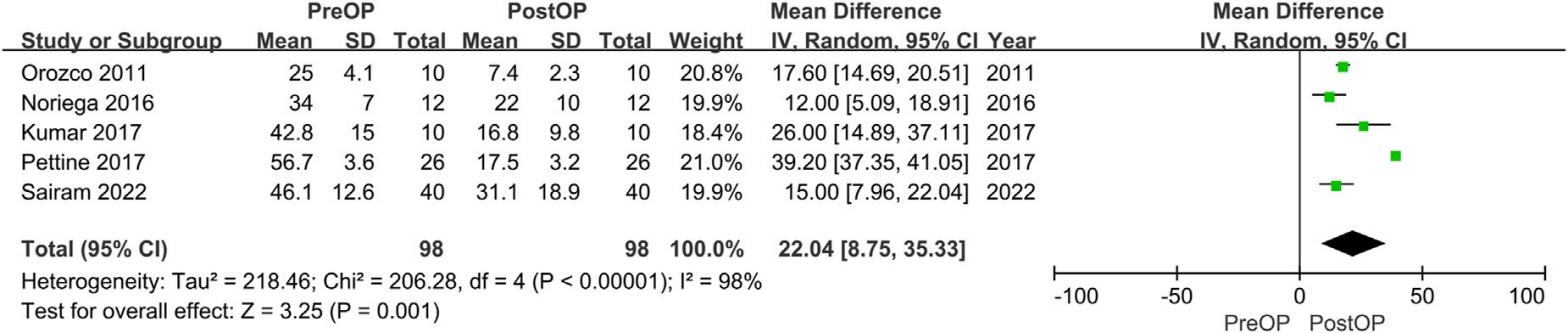
Forest plot for the ODI.

Four studies reported the data of ODI without stating the standard deviation. The remaining 5 studies could be further pool-analyzed and demonstrated that MSCs could improve Oswestry Disability Index significantly in patients with lumbar discogenic pain (mean difference = 22.04; 95% confidence interval [CI] 8.75 to 35.33; *p* = 0.001; Heterogeneity: I^2^ = 98%; *p* < 0.001) (Orozco et al., 2011; Kumar et al., 2017; Noriega et al., 2017; Pettine et al., 2017; Atluri et al., 2022) (Figure 5).

**FIGURE 6:**
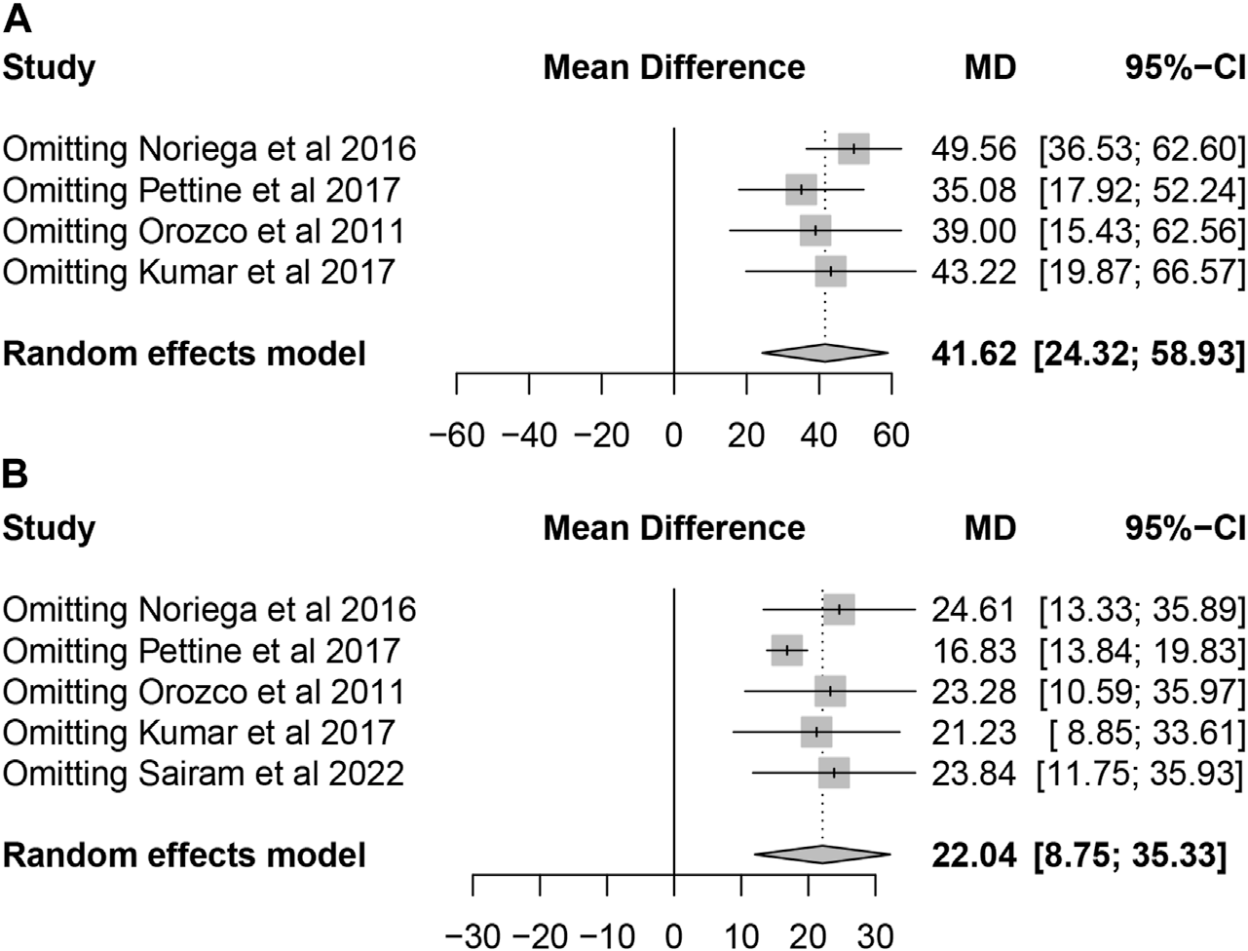
Sensitivity analysis by leave one out for **(A)** VAS and **(B)** ODI.

All included studies confirmed that patients with lumbar discogenic pain showed a trend of significant improvement in pain scores and ODI after treatment with MSCs. Sensitivity analysis was carried out by omitting studies one by one which demonstrated that the results were stable (Figure 6).

### 3.4 Secondary outcomes

**FIGURE 7:**
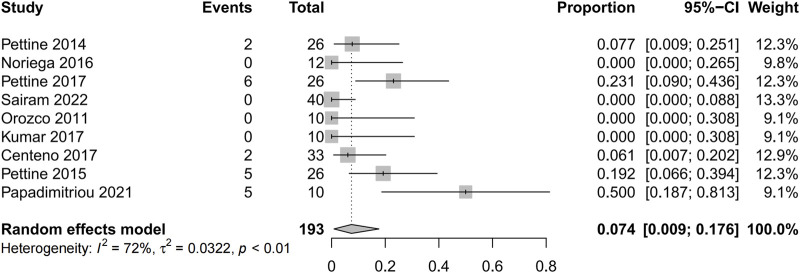
Forest plot for the reoperation proportion.

Five studies reported the reoperation proportion (20/193 [10.4%]) and the pooled analysis showed that after the application of mesenchymal stem cells was associated with extremely low percentage of reoperations (0.074 95% confidence interval [CI] 0.009 to 0.175; Heterogeneity: I2 = 72%; *p* < 0.01) (Pettine et al., 2014; Pettine et al., 2015; Centeno et al., 2017; Pettine et al., 2017; Papadimitriou et al., 2021) (Figure 7). In this study, while 20 patients ultimately opted for surgical treatment, there was still a proportion of patients who reported unsatisfactory surgical outcomes, indicating the uncertainty of surgical treatment for discogenic pain. No adverse events were reported in any of the studies.

## 4 Discussion

Intervertebral disc (IVD) degeneration is the leading cause of low back pain, a prevalent chronic condition with a significant impact on quality of life (Wu et al., 2020). The changes in the microenvironment of degenerative disc disease accompany a series of obstacles that hinder successful therapeutic intervention (Binch et al., 2021). The treatment of disc degeneration with novel cell-based therapies involves the transportation of living cells to the nucleus pulposus region to repopulate and repair the damaged disc to regulate the degenerative environment. This treatment is now gaining a great deal of attention (Eve et al., 2008). However, the evidence to support its use in clinical practice is still unclear. Influential clinical studies deserve prominent attention. To our knowledge, this is the first systematic analysis conducted to reevaluate these important findings. The key consideration of the current study was to collect and analyze the published influential clinical studies focusing on the effectiveness of the intra-articular injection of mesenchymal stem cells (MSCs) for the treatment of intervertebral disc (IVD) degeneration in a rigorous and comprehensive manner. The results showed and verified that the VAS and ODI scores were significantly lower in patients after receiving MSCs therapy. In addition, the scarcity of comorbidities and reoperation events does illustrate the safety of MSCs for the low back pain caused by IVD.

With the development of cell and tissue engineering techniques, MSCs from different sources such as bone marrow, adipose tissue, synovial membrane and skeletal muscle are widely used in the treatment and research of various orthopaedic diseases. The use of MSCs for the treatment of osteoarthritis is now in clinical research, and bone marrow and adipose-derived MSCs have shown great therapeutic potential in relieving joint pain and improving mobility through immunomodulatory repair and cartilage reconstruction (Lamo-Espinosa et al., 2018; Lee et al., 2019; Lu et al., 2019; Wang et al., 2020). While it is difficult to inject MSCs into the patient’s osteoporotic site in clinical work and has little clinical utility, relevant animal studies have demonstrated the high osteogenic differentiation capacity of MSCs and have the potential to be used as a therapeutic treatment for osteoporosis in future clinical work (Ye et al., 2014; Wang et al., 2017). In addition, MSCs have also shown promising clinical outcomes in the treatment of microfractures and bone defects.

Several cell types have been investigated for the management of IVD degeneration. Disc chondrocytes, such as activated nucleus pulpous (NP) cells, have been successfully isolated from intervertebral disc tissue, expanded in culture and used as a treatment for disc degeneration (Risbud et al., 2015). For patients receiving disc surgery, cells may need to be collected from adjacent discs to obtain adequate cell counts. The high quality of the disc matrix and the maintenance of disc cell populations are essential to inhibit disc degeneration. However, the reproduction capacity of the disc cells themselves is very low (Watanabe et al., 2010). Transplantation of NP cells alone may not be sufficient to suppress intervertebral disc degeneration. Mesenchymal stem cells (MSCs) show exciting promise in intervertebral disc repair strategies. MSCs can be isolated from many tissues, including bone marrow, adipose tissue and synovium (Oehme et al., 2015). The cell sources involved in the 9 influential clinical studies identified in this study all concerned the clinical effectiveness and safety of bone marrow derived MSCs (BMDSCs) in the treatment of IVD degeneration. BMDSCs not only differentiate into nucleus pulposus cells themselves but also nourish the remaining NP cells by producing cytokines such as transforming growth factor-β1 (TGF-β1) (Wang et al., 2010; Shim et al., 2016). However, since BMSCs account for only a small proportion of bone marrow cells tissue damage is inevitable during the extraction process. In contrast, adipose-derived stem cells (ADSCs) can be easily collected from adipose tissue with a higher yield and acceptance (Schmitt et al., 2021). Mesenchymal stem cells (MSCs) derived from the micro-fragmented adipose tissue (MFAT) have strong anti-inflammatory properties. MFAT as a tissue graft can liberate MSCs for a considerable period of time and its injection application can reduce pain and may promote tissue regeneration and repair (Vezzani et al., 2018; Nava et al., 2019). Relevant studies have demonstrated that intraarticular injection of MFAT has yielded beneficial clinical results, but its use in discogenic pain is still in the preliminary stages of exploration (Ferracini et al., 2022; Natali et al., 2022). The ADSCs were not included in our study, which may be subject to cell source bias.

Pain management and evaluation have always been a priority for treatment and are seen as indicators of credible effectiveness (Thong et al., 2018). The VAS is the most commonly used scoring system in pain evaluation. Only 4 studies included the complete VAS in our analysis but with a significant extent of pain improvement after BMSCs injection. Xie B et al. (Xie et al., 2021) conducted a systematic review and meta-analysis of clinical trials on the clinical efficacy and safety of MSCs for IVD degeneration and found that MSCs therapy could significantly decrease VAS scores (SMD = −0.50, 95%CI = −0.68 ∼ −0.33, *p* < 0.00001), compared with the control group. A more significant change in pain was reported in our study (SMD = 41.62, 95% CI = 24.32–58.93, Heterogeneity: I2 = 98%; *p* < 0.01) (the outcomes were expressed using a 0%–100% scale in our studies). In addition, the ODI allows an accurate and reliable assessment of the outcome of patients with chronic lower back pain and assessment of lumbar spine dysfunction by IVD degeneration (Sandal et al., 2021). The same improvement extent in the oswestry disability index (ODI) score was also reported (SMD = −0.27, 95% CI = −0.44 ∼ −0.09, *p* = 0.003) vs. our study (SMD = 22.04, 95% CI = 8.75–35.33, *p* < 0.0001). To sum up, the results of this meta-analysis suggest that bone marrow derived MSCs (BMDSCs) injection can significantly reduce pain and functional degeneration in patients with IVD degeneration.

MSCs characterized by low immunogenicity, easy access, and immunosuppressive potential, which make them strong attractive and application prospects (Kim and Cho, 2016). Even so, safety assessments need to be a priority. Studies have shown that the quality of MSCs depends more on the age of the donor, genetic characteristics, cell isolation conditions, and cell culture techniques (Pachon-Pena et al., 2016; Lukomska et al., 2019). Adverse events (AE) of treatment-emergent adverse events (TEAE) mainly include back pain, arthralgia, and muscle spasms. Previously published meta-analyses have shown that the MSCs injection does not produce statistically and clinically significant adverse events (Wu et al., 2018; Xie et al., 2021). Accordingly, the current study also showed that there was no significant AE of BMDSCs transplantation for patients with IVD degeneration. In addition, the reoperation rate was also low (0.74%).

This study has limitations. First, we found slight differences in the form of reporting of VAS and ODI across studies. Although 9 clinical studies were included in our study, only four and five studies were used to analyze VAS and ODI, respectively. This may underestimate the clinical significance of the study results. Second, our study only analyzed the clinical effectiveness and safety of BMSCs and lacked generalization to other MSCs, such as ADSCs. Third, the sample size of our study was relatively small (of the 245 patients, 193 received injections of BMSCs). Large and long-term clinical studies are still urgently needed.

## 5 Conclusion

The findings of our present systematic review and meta-analysis showed that mesenchymal stem cells (MSCs) injection therapy may be effective in relieving discogenic low back pain and improving Oswestry Disability Index significantly in patients with IVD degeneration. MSCs therapy seems to be a safe and effective alternative for the treatment of discogenic low back pain. Large scale studies and further RCT studies are warranted to better clarify the role of MSCs therapy in treating discogenic pain.

## Data Availability

The original contributions presented in the study are included in the article/Supplementary Material, further inquiries can be directed to the corresponding authors.

## Abbreviations

MSCs, smesenchymal stem cells; IVD, intervertebral disc; ODI, Oswestry Disability Index; RR, Risk ratios; VAS, Visual Analogue Scale; CI, confidence interval; WMD, weighted mean difference.
