# Supplementary material for: Mesenchymal stem cells can improve discogenic pain in patients with intervertebral disc degeneration: a systematic review and meta-analysis
Source: Front Bioeng Biotechnol. 2023 Jun 16;11:1155357. doi: 10.3389/fbioe.2023.1155357 (PMC10313064; doi:10.3389/fbioe.2023.1155357)
Supplement: Supplementary file 1 [file Table1.DOCX]

**Supplementary material:**

**Literature search strategy and exclusion criteria:**

**This search strategy follows the PICOS principles:**

**P:** patients with Intervertebral disc (IVD) degeneration-related diseases such as low back pain (LBP), lumbar disc degeneration, etc.

**Search strategy:**

1. Intervertebral Disc Degeneration*, Disc Degeneration*, Degenerative Disc Disease*, Degenerative Intervertebral Disc*, Degenerative Intervertebral Disk*.
2. Low back pain*, low backache*, low back ache*.

**I:** cell-based therapies mainly focus on mesenchymal stem cells (MSC)

**Search strategy:**

Mesenchymal Stem Cell*, Bone Marrow Stromal Cell*, Mesenchymal Stromal Cell*, Mesenchymal Progenitor Cell*

**C:** None

**O:** efficacy and safety of MSCs

**S:** clinical trials or influential clinical studies

**Table 1-4 details the search strategy used in the Medline, Embase, web of science, and Cochrane library databases.**

**Table 1: Literature search strategy in PubMed**

| Database platform | **PubMed** | |
| --- | --- | --- |
| Database | MEDINA | |
| Date of search | Sept 18, 2022 | |
| Limiters | No | |
| Search terms | | Results |
| #1 ((("Intervertebral Disc Degeneration"[Mesh]) OR ("Low Back Pain"[Mesh])) OR (Intervertebral Disc Degeneration* OR Disc Degeneration* OR Degenerative Disc Disease* OR Degenerative Intervertebral Disc* OR Degenerative Intervertebral Disk*)) OR (Low back pain* OR low backache* OR low back ache*) | | 59,724 |
| #2 ("Mesenchymal Stem Cells"[Mesh]) OR (Mesenchymal Stem Cell* OR Bone Marrow Stromal Cell* OR Mesenchymal Stromal Cell* OR Mesenchymal Progenitor Cell*) | | 106,213 |
| #3 #1 AND #2 | | 618 |

**Table 2: Literature search strategy in Embase**

| Database platform | **Embase** | |
| --- | --- | --- |
| Database | MEDINA and Embase | |
| Date of search | Sept 18, 2022 | |
| Limiters | No | |
| Search terms | | Results |
| #1 'intervertebral disk degeneration'/exp OR 'low back pain'/exp OR ‘Intervertebral Disc Degeneration’ OR ‘Disc Degeneration’ OR ‘Degenerative Disc Disease’ OR ‘Degenerative Intervertebral Disc’ OR ‘Degenerative Intervertebral Disk’ OR ‘Low back pain’ OR ‘low backache’ OR ‘low back ache’ | | 99,725 |
| #2 'mesenchymal stem cell'/exp OR 'bone marrow stroma cell' OR 'mesenchymal stroma cell' OR 'mesenchymal stem cell' | | 102,464 |
| #3 #1 AND #2 | | 879 |

**Table 3: Literature search strategy in web of science**

| Database platform | **web of science** | |
| --- | --- | --- |
| Database | SCI/SCIE | |
| Date of search | Sept, 18, 2022 | |
| Limiters | No | |
| Search terms | | Results |
| #1 TS=(Intervertebral Disc Degeneration* OR Disc Degeneration* OR Degenerative Disc Disease* OR Degenerative Intervertebral Disc*OR Degenerative Intervertebral Disk*OR Low back pain* OR low backache* OR low back ache*) | | 14,634 |
| #2 TS=(Mesenchymal Stem Cell* OR Bone Marrow Stromal Cell* OR Mesenchymal Stromal Cell* OR Mesenchymal Progenitor Cell*) | | 136,328 |
| #3 #1 AND #2 | | 859 |

**Table 4: Literature search strategy in Cochrane library**

| Database platform | **Cochrane library** | |
| --- | --- | --- |
| Database | **Cochrane library** | |
| Date of search | Sept, 18, 2022 | |
| Limiters | No | |
| Search terms | | Results |
| #1 [Intervertebral Disc Degeneration] explode all trees OR [Low Back Pain] explode all trees OR (Intervertebral Disc Degeneration):ti,ab,kw OR (Disc Degeneration):ti,ab,kw OR (Degenerative Disc Disease):ti,ab,kw OR (Degenerative Intervertebral Disc):ti,ab,kw OR (Degenerative Intervertebral Disk):ti,ab,kw OR (low back pain):ti,ab,kw OR (low backache):ti,ab,kw OR (low back ache):ti,ab,kw | | 15,107 |
| #2 [Mesenchymal Stem Cell Transplantation] explode all trees OR (Mesenchymal Stem Cell):ti,ab,kw OR (Bone Marrow Stromal Cell):ti,ab,kw OR (Mesenchymal Stromal Cell):ti,ab,kw OR (Mesenchymal Progenitor Cell):ti,ab,kw | | 2,187 |
| #3 #1 AND #2 | | 36 |
